# Investigation of size–dependent cell adhesion on nanostructured interfaces

**DOI:** 10.1186/s12951-014-0054-4

**Published:** 2014-12-05

**Authors:** Chiung Wen Kuo, Di-Yen Chueh, Peilin Chen

**Affiliations:** Research Center for Applied Sciences, Academia Sinica, 128, Section 2, Academia Road, Nankang, Taipei 11529 Taiwan

**Keywords:** Nanotopography, Cell adhesion, Surface topography

## Abstract

**Background:**

Cells explore the surfaces of materials through membrane-bound receptors, such as the integrins, and use them to interact with extracellular matrix molecules adsorbed on the substrate surfaces, resulting in the formation of focal adhesions. With recent advances in nanotechnology, biosensors and bioelectronics are being fabricated with ever decreasing feature sizes. The performances of these devices depend on how cells interact with nanostructures on the device surfaces. However, the behavior of cells on nanostructures is not yet fully understood. Here we present a systematic study of cell-nanostructure interaction using polymeric nanopillars with various diameters.

**Results:**

We first checked the viability of cells grown on nanopillars with diameters ranging from 200 nm to 700 nm. It was observed that when cells were cultured on the nanopillars, the apoptosis rate slightly increased as the size of the nanopillar decreased. We then calculated the average size of the focal adhesions and the cell-spreading area for focal adhesions using confocal microscopy. The size of focal adhesions formed on the nanopillars was found to decrease as the size of the nanopillars decreased, resembling the formations of nascent focal complexes. However, when the size of nanopillars decreased to 200 nm, the size of the focal adhesions increased. Further study revealed that cells interacted very strongly with the nanopillars with a diameter of 200 nm and exerted sufficient forces to bend the nanopillars together, resulting in the formation of larger focal adhesions.

**Conclusions:**

We have developed a simple approach to systematically study cell-substrate interactions on physically well-defined substrates using size-tunable polymeric nanopillars. From this study, we conclude that cells can survive on nanostructures with a slight increase in apoptosis rate and that cells interact very strongly with smaller nanostructures. In contrast to previous observations on flat substrates that cells interacted weakly with softer substrates, we observed strong cell-substrate interactions on the softer nanopillars with smaller diameters. Our results indicate that in addition to substrate rigidity, nanostructure dimensions are additional important physical parameters that can be used to regulate behaviour of cells.

## Background

The interfacial properties of materials govern the performance of biomaterials because cells are in direct contact with the surfaces of materials. Cells explore the surfaces of materials through membrane-bound receptors, such as the integrins, and use them to interact with extracellular matrix (ECM) molecules adsorbed on the substrate surfaces, resulting in the formation of focal adhesions [1-6]. Therefore, one of the commonly used approaches to improve the performance of biomaterials is surface engineering, whereby a material’s surface properties can be modified by chemical and physical means. In the past few decades, surface engineering techniques have been widely used to improve device biocompatibility, to promote cell adhesion and to reduce unwanted protein adsorption [7-13]. With recent advances in nanotechnology, biosensors and bioelectronics are being fabricated with ever decreasing feature sizes. The performances of these devices depend on how cells interact with nanostructures on the device surfaces. However, the behavior of cells on nanostructures is not yet fully understood.

To investigate how cells respond to their nanoenvironments, many techniques, including dip-pen lithography [14], electron-beam lithography [15], nano-imprinting [16], block-copolymer micelle nanolithography [17-21], and nanosphere lithography [22], have been utilized to create well-defined protein nanopatterns on planar substrates. The dimensional parameters of ECM molecules, including density, spacing, and surface coverage, have been found to be important to cell adhesion and spreading. When cells attach to surfaces, nanometer-scale dot-like focal complexes are formed first [5]. These focal complexes are transient and unstable. Some of the focal complexes will mature into micrometer-scale elongated focal adhesions, which serve as anchoring points for cells. It has been previously shown [22,23] that the formation of focal adhesions was dependent on the size of the ECM nanopatterns and that the force experienced by the focal adhesions increased as the pattern size decreased, explaining the instability of smaller focal complexes.

In addition to sensing the protein composition of the nanoenvironment, cells also sense the physical properties around them. It has been demonstrated that by systematically changing the rigidity of microstructures, the regulation of cell functions, such as morphology, focal adhesions and stem cell differentiation, can occur [24]. It was recently observed that the efficiency of drug-uptake by cells was greatly enhanced for cells grown on nanostructured materials, including roughened polymers [25], nanowires [26], nanofibers [27] and nanotubes [28,29]. However, the mechanisms by which the cells interact with these nanostructures have not yet been studied systematically [30-32]. To understand how cells interact with nanostructures, we have systematically investigated the interactions between cells and nanostructures using size-tunable polymeric nanopillars with well-defined physical properties.

## Results and discussion

**Figure 1:**
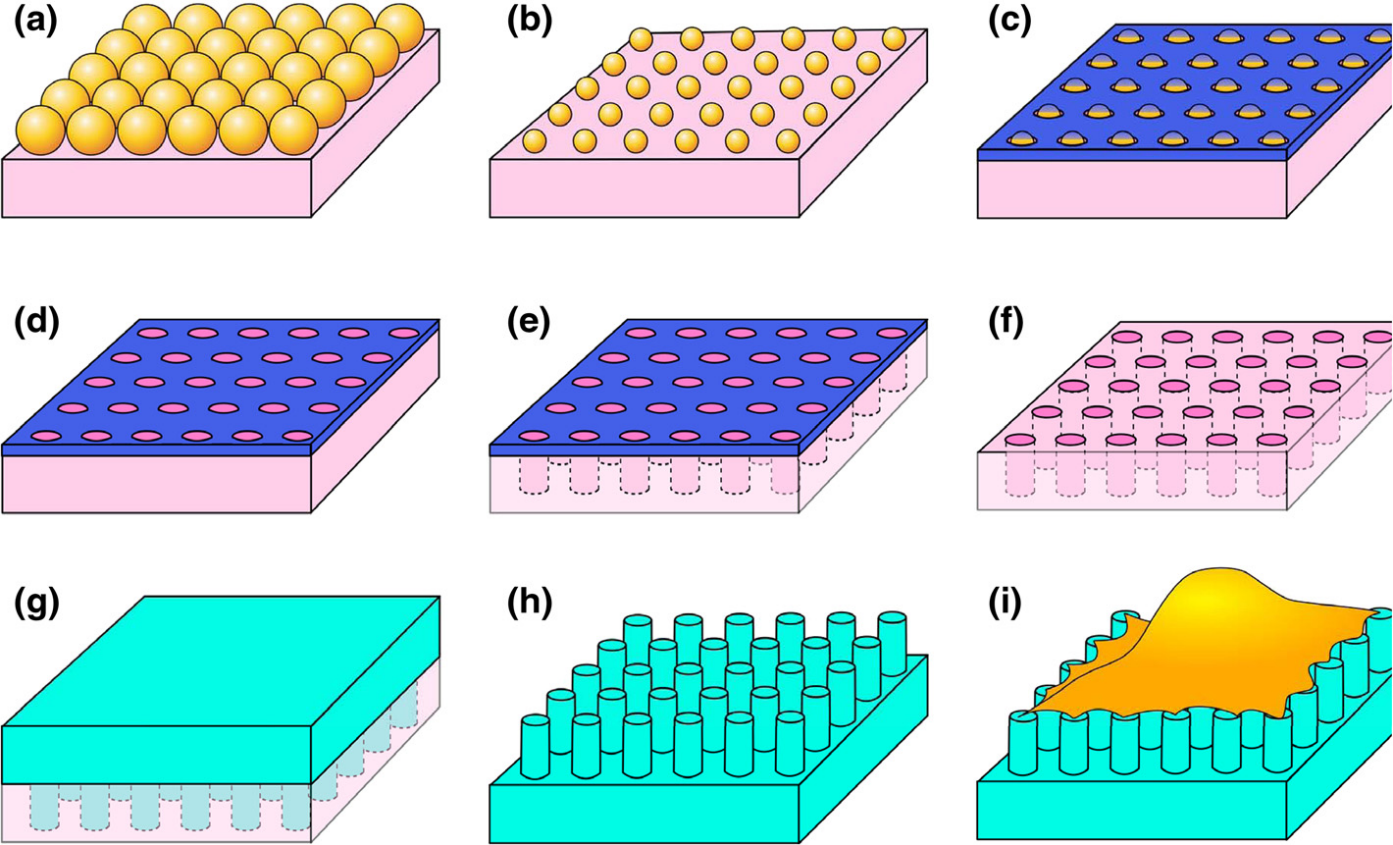
**A schematic representation of the fabrication of the polymeric nanopillar arrays. (a)** Nanosphere lithography is utilized to produce a single-layer closely packed structure. **(b)** The diameters of the polystyrene beads are reduced to a specific size by the oxygen-plasma process. **(c)** A layer of nickel is evaporated on the top of the polystyrene beads. **(d)** The polystyrene beads are dissolved with dichloromethane. **(e)** The nickel film serves as the etching mask for the silicon-hole array in ICP etching. **(f)** The nickel film is removed by the etchant solution. **(g)** The silicon-hole arrays are used as molds for replication by spin-coating the photo-curable polymers. The UV-curable adhesive is cured under UV radiation for 10 minutes. **(h)** After being peeled away from the silicon mold, well-ordered periodic polymeric nanopillar arrays are obtained. **(i)** The polymeric nanopillar arrays are then used to culture cells.

**Figure 2:**
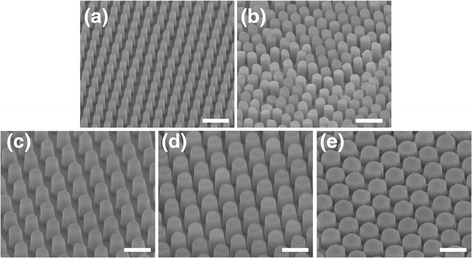
**Scanning electron micrographs of polymeric nanopillar arrays made of UV-curable adhesive.** The diameters of the nanopillars are **(a)** 214 ± 13 nm, **(b)** 322 ± 16 nm, **(c)** 425 ± 17 nm, **(d)** 500 ± 19 nm, and **(e)** 684 ± 17 nm. Scale bars are 1 μm.

In recent years, nanosphere lithography has been utilized to fabricate well-ordered periodic nanostructures over large areas [33,34]. In this experiment, nanosphere lithography was employed to fabricate nanohole arrays to be used as replication masters, which were then used to produce nanopillars with various dimensions, as shown in Figure 1. Several curable polymers, such as PDMS, h-PDMS, PMMA, Teflon and SU-8 photo-resist, have been used to replicate the nanostructure of the silicon nanohole arrays. In this experiment, we selected a UV-curable adhesive (NOA 61, Norland) to produce the nanopillars due to the simplicity of its use in fabrication. Figure 2 presents SEM images of size-tunable polymeric nanopillar arrays made of UV-curable adhesive. The diameters of the nanopillars ranged from 200 nm to 700 nm, and their heights ranged from 700 nm to 1000 nm. The measured diameters and heights of the fabricated nanopillars are listed in Table 1, as well as the calculated rigidity, which decreased from 94 nN/nm for the nanopillars 680 nm in diameter and 660 nm in height to 0.26 nN/nm for the nanopillars 200 nm in diameter and 800 nm in height. The biocompatibility of the polymeric nanopillars could be improved by coating their surfaces with a layer of ECM molecules, such as fibronectin.

**Table 1:** **Dimensions and apparent Young’s moduli measured for nanopillars**

**Spacing (nm)**	**Diameter (nm)**	**Height (nm)**	**Young’s modulus (MPa)**	**Rigidity (nN/nm)**
540	214 ± 13	781 ± 37	402 ± 130	0.26
540	322 ± 16	999 ± 88	583 ± 128	0.93
870	425 ± 17	814 ± 56	697 ± 118	6.21
870	500 ± 19	971 ± 55	721 ± 164	7.24
870	684 ± 17	663 ± 49	852 ± 249	94.19

**Figure 3:**
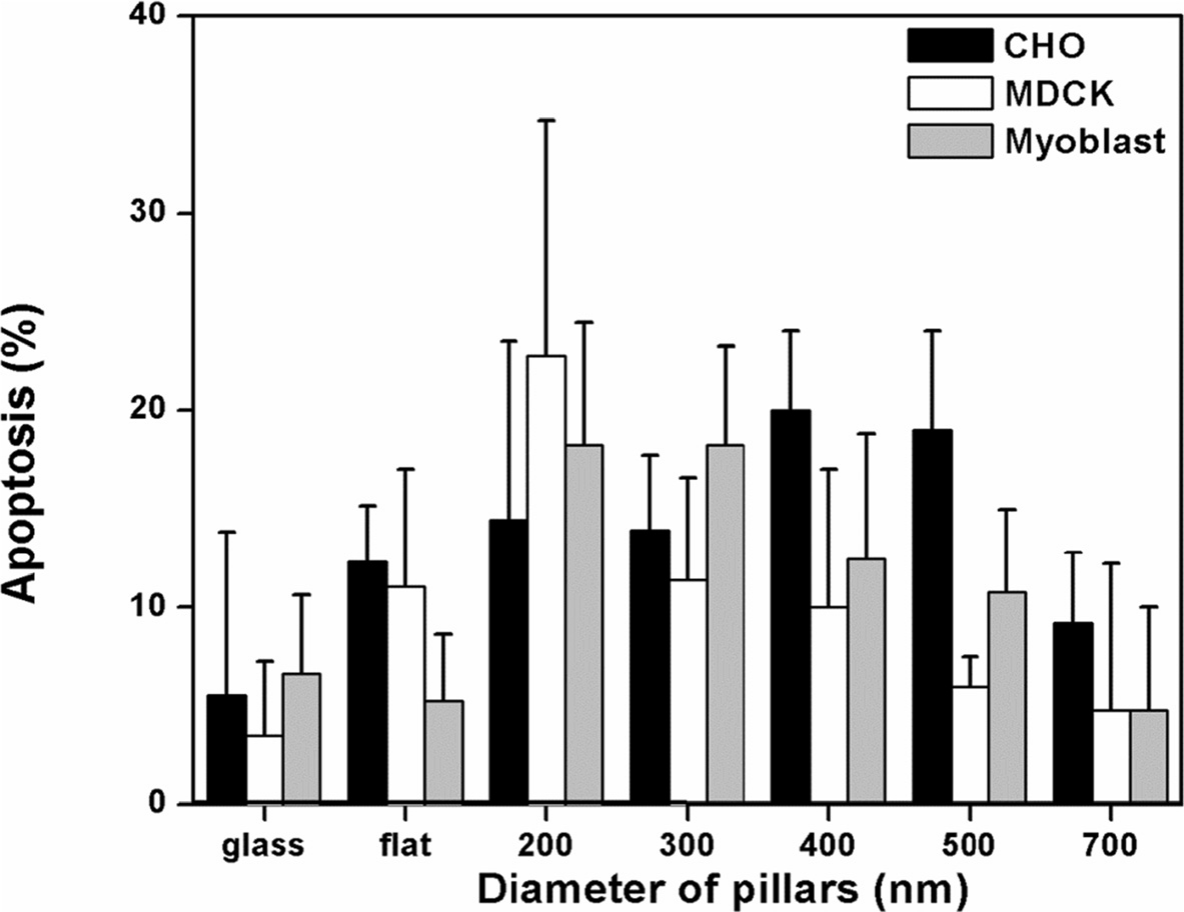
**Apoptotic cells (percentage) after 24 hours of incubation on nanopillar arrays of varied diameters and controls.**

When cells are cultured on structures with geometric constraints, the dimensions of the structures may influence the behavior of the cells. It has been shown that cells undergo apoptosis when confined to micro-patterns with dimensions less than 10 μm [10]. However, the responses of cells grown on nanopillars with a separation distance less than 1 μm are less studied. Therefore, we first evaluated the viability of cells on nanopillars with various diameters using an apoptosis assay. Three cell lines CHO, MDCK, and C2C12, were used in this experiment because of their applications in biotechnology and pharmaceutical industries [35]. These three cells are often used in the studies of cell adhesions because they form prominent focal adhesions. CHO MDCK cells are epithelial cells whereas the C2C12 cells are myoblast. The results presented in Figure 3 show the percentage of cells that underwent apoptosis when grown on nanopillar substrates with diameters ranging from 200 nm to 700 nm. As a general observation, the percentage of cells that underwent apoptosis was increased slightly for cells cultured on the nanopillars. As the diameter of the nanopillars decreased from 700 nm to 200 nm, the percentage of cells that underwent apoptosis increased from approximately 5% to approximately 20% for all cell lines tested, indicating that the viability of cells was slightly compromised on the smaller nanostructures.

**Figure 4:**
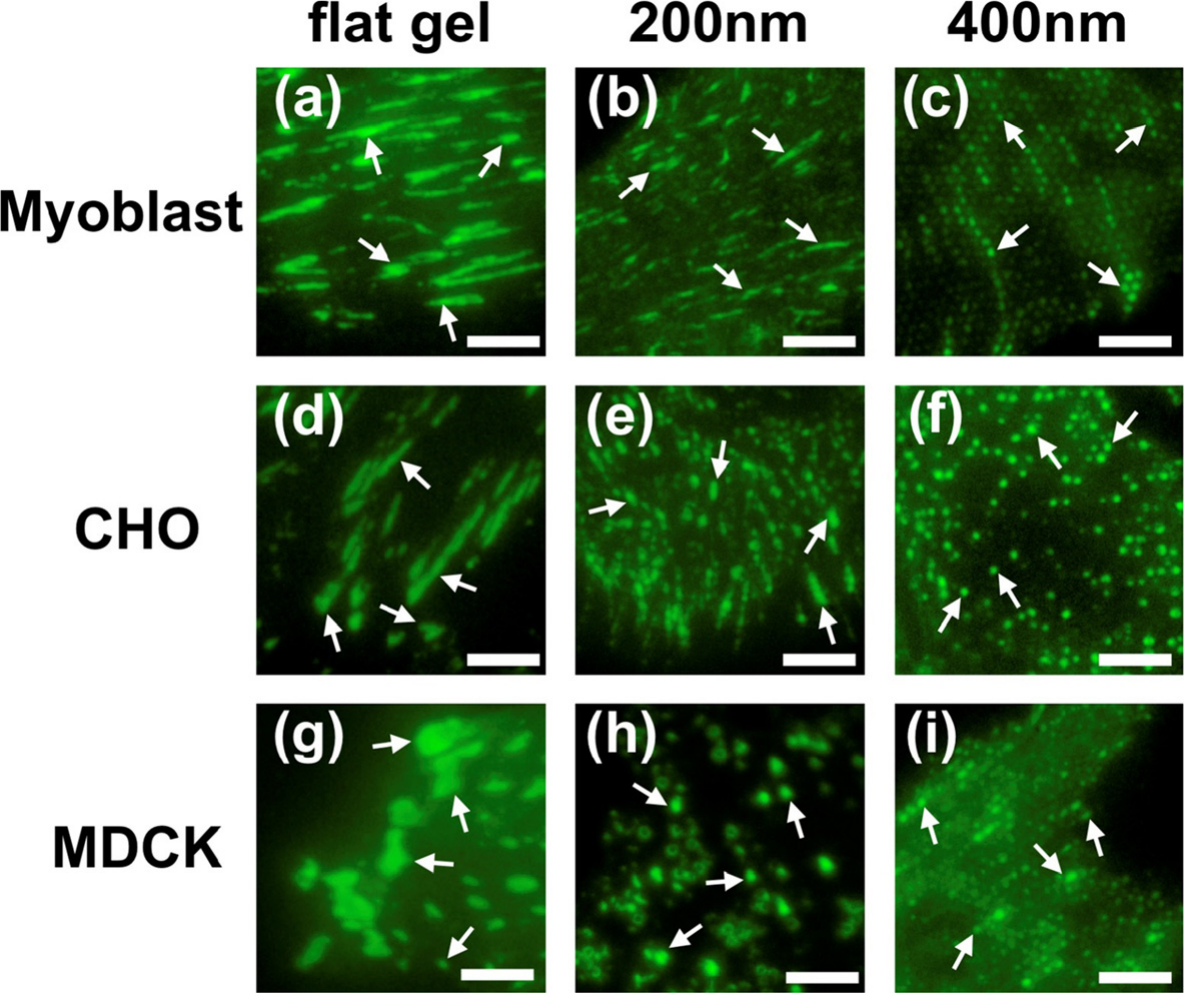
**Confocal images of focal adhesions formed on (a), (d), (g) the flat NOA 61 substrates, (b), (e), (h) 200 nm nanopillars and (c), (f), (i) 400 nm nanopillars for myoblast, CHO and MDCK cells, respectively.** Green coloration indicates the location of vinculin proteins. Arrows indicate clustering of vinculin in the focal adhesions. Scale bar is 5 μm.

To understand how cells interact with the nanopillars, we used confocal microscopy to investigate the formation of focal adhesions by staining vinculin, a major component of the focal adhesion complex. Figure 4 presents confocal images of focal adhesions formed on the flat substrates and the nanopillars. It is known that cells explore their microenvironments through surface receptors such as integrins. When integrin molecules bind to ECM molecules, they recruit various focal adhesion molecules, including vinculin, paxillin, and talin, among others, to form sub-micrometer-scale focal complexes [5]. These focal complexes then mature to form elongated focal adhesions if the microenvironments surrounding the cells are suitable for cell adhesion. These processes are very dynamic, and the focal complexes are not stable. In Figure 4a,d and g, the formation of elongated focal adhesions on the flat substrate can be clearly observed. However, when cells were cultured on top of the nanopillars, the size of the focal adhesions was confined to the size of the nanopillars. These adhesions, which resemble focal complexes, are shown in Figures 4c,f and i. When the diameter of the nanopillars was further reduced to 200 nm, the shape of the focal adhesion was cell-line dependent. In the myoblast cells, large focal adhesions were observed, similar to those found on the flat substrates. In the CHO cells, some of the focal adhesions were dot-like and some were larger. In the MDCK cell lines, larger dots were observed. In general, the focal adhesions formed on the 200-nm nanopillars were larger than those formed on the 400-nm nanopillars.

**Figure 5:**
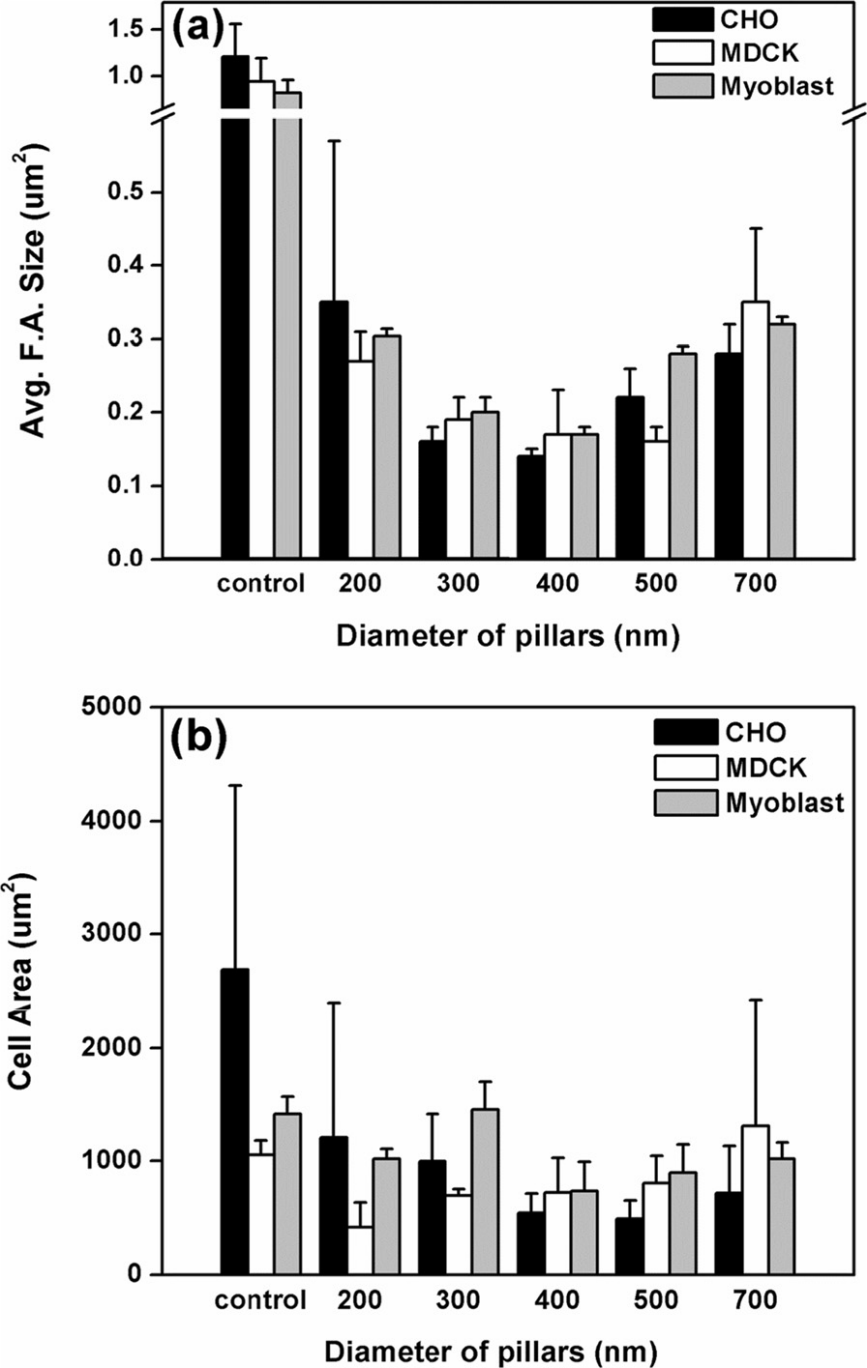
**Measured focal adhesion sizes and cell spreading areas on nanopillars. (a)** Size of focal adhesions; **(b)** area of cell spreading measured on various nanopillars. Data are presented as the means ± SD.

To quantify these results, we calculated the average size of the focal adhesions and the cell-spreading area for focal adhesions grown on nanopillars with diameters ranging from 200 nm to 700 nm. These results are summarized in Figure 5. The average area of the focal adhesions in the MDCK, myoblast and CHO cells grown on the nanopillars were measured to be between 0.2 and 0.4 μm^2^; all of these values were much smaller than the corresponding areas observed for cells grown on the flat substrates (which were larger than 1 μm^2^ as shown in Figure 4). The lengths of the focal adhesions formed on the UV-adhesive-coated flat substrates were 3.16 ± 0.35 μm, 2.39 ± 0.76 μm and 2.05 ± 0.57 μm for the myoblast, CHO and MDCK cells, respectively, and their corresponding widths were 0.66 ± 0.13 μm, 0.85 ± 0.13 μm and 0.93 ± 0.29 μm. On the 400-nm nanopillar, the measured lengths were 0.61 ± 0.08 μm, 0.56 ± 0.08 μm and 0.59 ± 0.07 μm for the myoblasts, CHO and MDCK cells, and the widths were 0.49 ± 0.06 μm, 0.54 ± 0.08 μm and 0.49 ± 0.07 μm. These widths were roughly the same as the size of the nanopillars. In other words, the focal adhesions formed on the nanopillars possessed a round shape rather than an elongated shape. Surprisingly, larger focal adhesions were observed on the 200-nm nanopillars, with lengths of 1.82 ± 1.05 μm, 1.45 ± 0.42 μm and 0.82 ± 0.24 μm and widths of 0.45 ± 0.08 μm, 0.58 ± 0.10 μm and 0.57 ± 0.09 μm for the myoblast, CHO and MDCK cells, respectively. The smallest focal adhesions were observed on the nanopillars with diameters of 400 nm (corresponding to a surface area of approximately 0.17 μm^2^); these adhesions resembled the nascent focal complexes. The nascent focal complexes were found to locate preferentially in the lamellipodium [4,5], consistent with an earlier finding that actin, talin and vinculin molecules were abundant in the small nascent focal complexes (< 1 μm^2^). From the data presented in Figure 5a, it is clear that the size of the focal adhesions decreased with the diameter of the nanopillars due to the spatial confinement of the nanopillars, which prevented further maturation of the focal adhesions. As a result of this spatial confinement, the number of focal adhesions increased and the total focal-adhesion area decreased as the size of nanopillars decreased. In general, the total cell-spreading area was smaller on the nanostructured surfaces compared to the flat surfaces and decreased as the size of nanopillars decreased, with the exception of cells cultured on the 200- and 300-nm nanopillars, on which larger focal adhesions were observed. It has been shown that cells in smaller focal adhesions exert stronger forces [22]. As small focal complexes mature into larger focal adhesions, cells exert contractile forces upon the substrates. In contrast to small focal complexes, the size of matured focal adhesions is generally larger than 1 μm^2^, and the size of a focal adhesion can be correlated directly with the magnitude of the traction force exerted by the cells [23,33]. Interestingly, when cells were cultured on the nanopillars, the spatial limitations of the nanopillars prevented the focal complexes from maturing to focal adhesions. As a result, the cells exerted considerable force on the smaller nanopillars, leading to bending of the nanopillars to form larger focal adhesions, as observed in the case of the 200-nm nanopillars. These findings agreed with our previous experiments in which nanopillars of 200 nm were used to measure the forces exerted by living CHO cells [36]; we observed forces of up to 40 nN exerted on the edges of cells.

**Figure 6:**
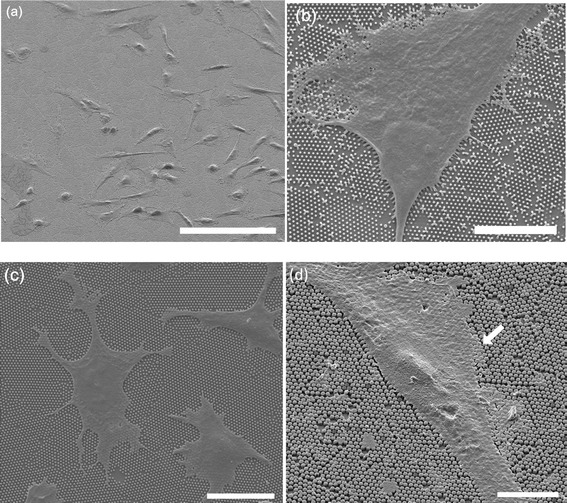
**SEM images of cells cultured on nanopillars. (a)** CHO cells cultured on 300-nm nanopillars. Scale bars are 100 μm. **(b)** MDCK cells on 200-nm nanopillars. Scale bars are 10 μm. **(c)** CHO cells cultured on 400-nm nanopillars, Scale bars are 20 μm. **(d)** CHO cells cultured on 700-nm nanopillars. Scale bars are 10 μm.

To further investigate the interaction between cells and nanostructures, we examined scanning electron microscope (SEM) images of cells cultured on nanopillars, which are shown in Figure 6. Figure 6a presents a large-area SEM image of cells cultured on 300-nm nanopillars. Two different morphologies can be observed: well-spread and elongated, both of which are typically observed in cells cultured on the nanopillars. Figure 6b shows cells that are well-spread on the 200-nm nanopillars. The contractile force exerted by the cells bent the nanopillars closer to each other. This bend is more pronounced at the edges of the cells, as shown in the lower part of the image. On the larger nanopillars (Figure 6c and d), the cells also exerted forces that were large in magnitude. However, the displacement of the larger nanopillars was not as significant as was observed with the 200-nm pillars because of the much higher rigidity of the larger nanopillar as seen in Table 1. It is generally accepted that cells cultured on stiff or rigid substrates promote more pulling and spreading, leading to an elongated morphology, while a round shape is often observed for cells cultured on soft substrates [37,38]. In this study, we observed stronger pulling and spreading on substrates with softer nanopillars. We attributed our observation to the use of smaller nanostructures where smaller focal complexes were first formed on the nanostructures leading to larger forces exerted by cells. Our experiment confirmed that the larger focal adhesion observed on the 200-nm nanopillars can be attributed to the larger traction force exerted by cells on the smaller nanopillars. It has been shown that the fate of stem cells can be regulated by modulating the rigidity of substrates using microposts of a uniform diameter but different lengths [14]. Here we demonstrated that the diameter of the nanopillars is another parameter that can be used to regulate the behavior of cells.

**Figure 7:**
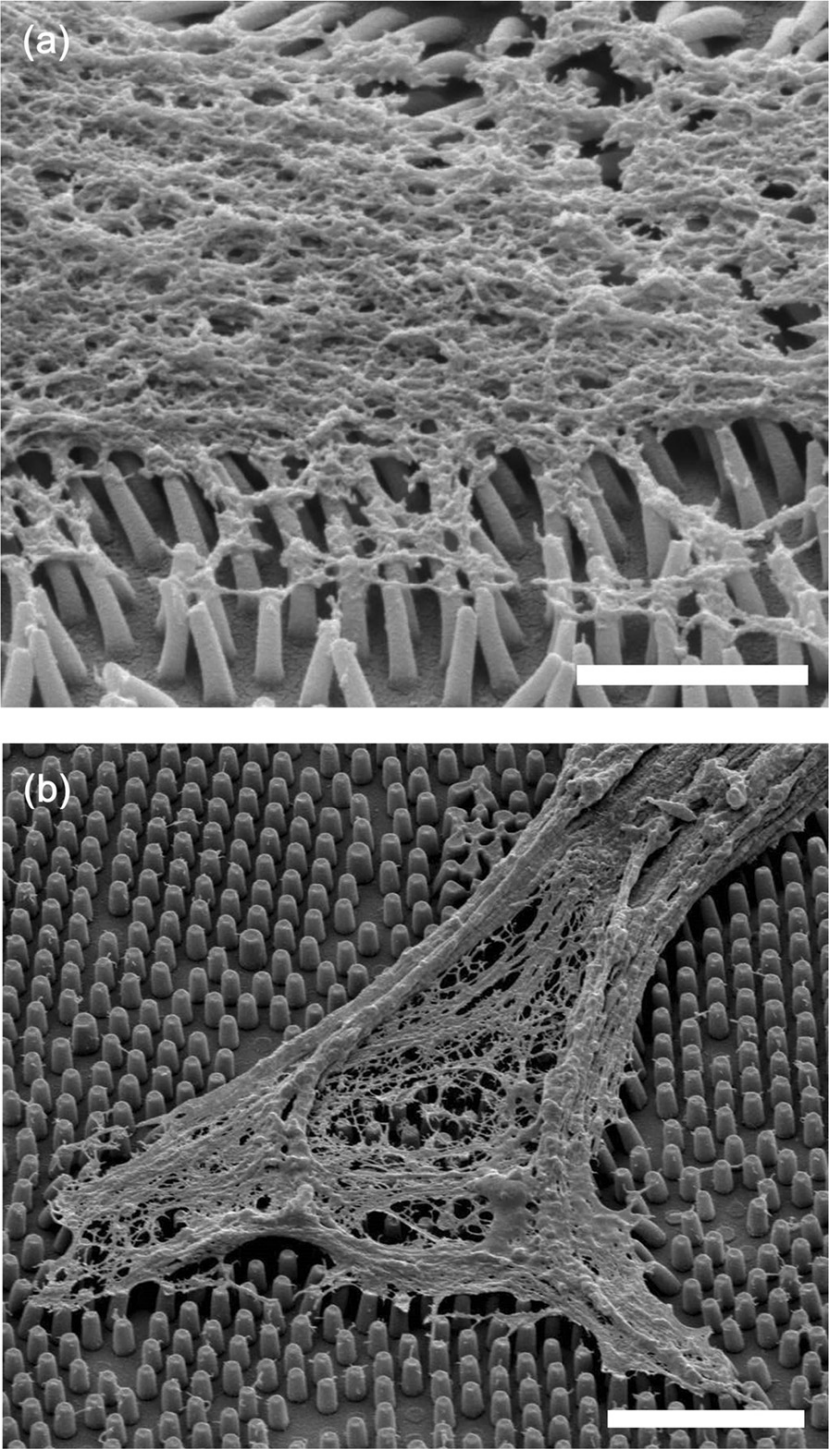
**SEM images of the internal cell structures after dissolution of the cell membranes. (a)** CHO cells cultured on 200-nm nanopillars Scale: 2 μm **(b)** CHO cells cultured on 400-nm nanopillars Scale bar: 5 μm.

Because the cells exert forces upon the focal adhesion through stress fibers, it is interesting to investigate how these stress fibers interact with the nanopillars. To clarify the role of actin fibers in the cell-substrate interaction, we dissolved the cell membranes with cytoskeleton buffers, revealing the internal structure of the actin filaments. In Figure 7a, we can see that when cells were cultured on the 200-nm nanopillars, the actin filaments pulled the nanopillars together very strongly and often bent them over. In contrast, on the 400-nm pillars, actin filaments interacted only slightly with the nanopillars, only bending them at the edges of the cells. This result further confirmed that the cells exerted stronger forces on the smaller nanopillars despite the fact that the rigidity of these nanopillars was lower.

**Figure 8:**
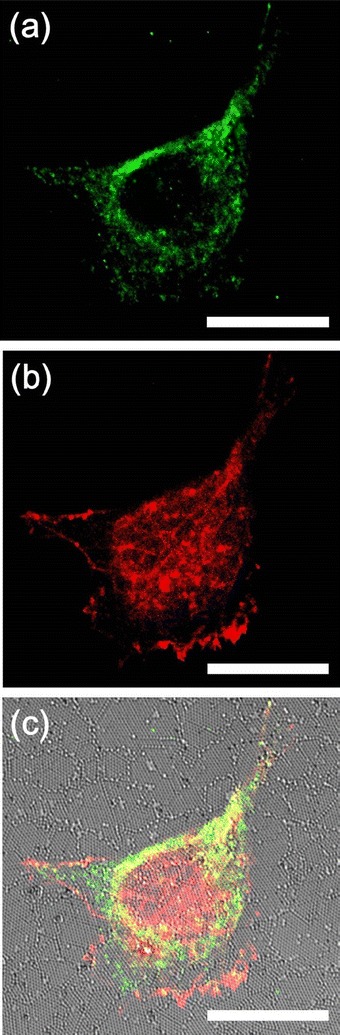
**Confocal image of a CHO cell cultured on a 200-nm nanopillar array immunofluorescently stained with (a) tensin (green) and (b) FAK (red) after 24 hours of incubation. (c)** Combined DIC and fluorescence image. Scale bar is 20 μm.

When cells were cultured on the 200-nm nanopillars, large focal adhesions were observed with confocal imaging, as seen in Figure 4. From the SEM images (Figure 6b, Figure 7a), we know that cells pulled the 200-nm nanopillars closer to each other. However, it was unclear whether the larger observed focal adhesions were due to the diffraction limit of the optical microscope, which prevents resolving adjacent smaller focal complexes or whether the focal adhesion indeed matured on the 200-nm nanopillars. To understand the state of the focal adhesions formed on the 200-nm nanopillars, we stained the cells with antibodies against focal adhesion kinase (FAK), a focal adhesion molecule recruited early to focal adhesions, and tensin, which is abundant in the matured fibrillar adhesion [3,39]. The results are summarized in Figure 8, with FAK illustrated in red and tensin with green. It can be noted that FAK is mainly located in the edge and center of the cell, whereas the tensin is abundant around the cell body. The degree of colocalization between these two molecules was relatively low. It is known that cells exert a larger force on the nascent focal adhesions, which are transient and unstable. As the focal complex matures to larger focal adhesions, the tension is reduced. As the focal adhesions evolve to fibrillar adhesions, they do not disassemble when the force is relaxed. On the 200-nm nanopillars, the focal complexes were first formed on the top of the nanopillars. The geometric limitation prevents the further maturation of the focal complex. However, the higher forces exerted on the 200-nm nanopillars with a significantly lower rigidity than the larger nanopillars led to the bending of the nanopillars, which allowed the maturation of focal adhesions. During this process, the cell membranes interact strongly with the surfaces of nanopillars enhancing the possibility for uptake of any drug molecules adsorbed on the surfaces as observed in several experiments [25-29].

## Conclusions

In summary, we have developed a simple approach to systematically study cell-substrate interactions on physically well-defined substrates using size-tunable polymeric nanopillars. When cells were cultured on the nanopillars, the apoptosis rate slightly increased as the size of the nanopillar decreased. The size of focal adhesions formed on the nanopillars decreased as the size of the nanopillars decreased, resembling the formations of nascent focal complexes. However, when the size of nanopillars decreased to 200 nm, the size of the focal adhesions increased. Further study revealed that cells interacted very strongly with the nanopillars with a diameter of 200 nm and exerted sufficient forces to bend the nanopillars together, resulting in the formation of larger focal adhesions. From this study, we conclude that cells can survive on nanostructures with a slight increase in apoptosis rate and that cells interact very strongly with smaller nanostructures. In contrast to previous observations on flat substrates that cells interacted weakly with softer substrates, we observed strong cell-substrate interactions on our softer nanopillars with smaller diameters. Our results indicate that in addition to substrate rigidity, nanostructure dimensions are additional important physical parameters that can be used to regulate cell behavior.

## Methods

### Fabrication of polymeric nanopillars

Polymeric nanopillars were fabricated via a combination of nanosphere lithography and nano-molding, as described previously [40]. The schematic for nanopillar fabrication is illustrated in Figure 1. Briefly, a closely packed monolayer of polystyrene beads with a diameter of 870 nm (Bang’s Laboratories, Inc., CV < 5%) was produced on the silicon substrates using nanosphere lithography, and the diameter of the polystyrene beads was reduced to various diameters of interest using oxygen plasma trimming. A layer of chromium was then deposited on the substrate. After removing the polystyrene beads using dichloromethane, a deep-etching process was performed in an inductively coupled plasma system (Samco, RIE-10ip). As a result of the deep-etching, periodic nanohole arrays were obtained on the silicon substrates, which were then used to create the polymeric nanopillars. To remove the chromium, silicon templates were placed in a chromium etchant (Aldrich) solution for 20 minutes. To produce the polymeric nanopillars, an ultraviolet-curable adhesive (NOA 61, Norland, Young’s modulus: 1.2 GPa) was spun onto the silicon-hole substrate at 1000 rpm and subsequently illuminated with UV light in an ELC-500 UV-light chamber for 10 minutes. The polymeric nanopillars were obtained by peeling off the cured films from the silicon-hole templates.

### Cell culture

CHO-K1 cells were seeded in 30-mm plates with Ham’s F-12 K medium supplemented with 10% (v/v) fetal bovine serum (FBS) and passaged every 2 to 3 days. MDCK cells were grown in minimum essential medium with Earle’s BSS supplemented with 10% (v/v) FBS, 2 mM L-glutamine, 0.1 mM non-essential amino acids, 1 mM sodium pyruvate and 1.5 g/L sodium bicarbonate. C2C12 myoblast cells were grown in Dulbecco’s modified Eagle’s medium supplemented with 10% (v/v) calf serum, 4 mM L-glutamine, 1.5 g/L sodium bicarbonate and 4.5 g/L glucose. PEN-STREP-AMPHO solution (Biological Industries) was added to all culture media. The cells were incubated at 37°C in a 5% CO_2_ atmosphere.

### Immunofluorescence staining

Cells were fixed by 4% (wt/vol) paraformaldehyde in phosphate-buffered saline (PBS) for 15 minutes, washed twice with PBS solution, and subsequently permeabilized in a 0.25% Triton X-100 solution for 20 minutes. Before staining, all of the samples were blocked using 1% BSA in PBS solution for 30 minutes. The samples were then incubated with the primary antibody at a concentration of 1 μg/mL for 2 hours. The samples were washed three times with PBS followed by incubation with a fluorescent-labeled anti-mouse secondary antibody for 1 hour. To visualize the cell morphology, actin filaments were labeled with TRITC-conjugated phalloidin. A Focal Adhesions Staining Kit (Chemicon) was used to label the focal adhesions. The fluorescence images were obtained using a Leica SP5 confocal microscope. The area of cell spreading and the size of the focal adhesions on the nanopillars of various diameters were analyzed using MetaMorph software (Universal Imaging).

### Apoptosis assay

To measure the apoptosis rates of cells grown on the nanopillars, three different cell lines (CHO, C2C12 and MDCK) were seeded on the substrates at a density of 1 × 10^5^ cells/mL and incubated at 37°C in a 5% CO_2_ atmosphere. Cells seeded on poly-L-lysine-coated and UV-adhesive-coated cover slips for 24 hours provided experimental controls. After 24 hours of culture, the cells were fixed in 4% paraformaldehyde in PBS for 15 minutes. Next, permeabilization was performed using a 20 minute incubation with 0.25% Triton X-100 followed by two washes with PBS. The TUNEL assay for apoptosis was used to measure DNA-damage fragmentation. The Click-iT TUNEL imaging assay (Invitrogen) utilized a dUTP modification with an alkyne group. Samples were incubated for 30 minutes at room temperature with 100 μL of the Click-iT reaction solution, after which the Click-iT reaction solution was removed and the samples were washed three times for 5 minutes with PBS containing 3% BSA. Finally, 100 μL of Hoechst 33342 solution was then added to the sample and incubated for 15 minutes for DNA staining.

### SEM images

To visualize cells on the nanopillars, the cells were fixed overnight at 4°C using a 2% (wt/vol) glutaraldehyde solution in 0.1 M sodium cacodylate, pH 7.3. Samples were then warmed to room temperature and washed twice with PBS. Before critical-point drying, the samples were incubated in 0.1% aqueous tannic acid for 20 minutes, after which the solution was gradually replaced with a PBS/ethanol mixture in the following progression of ratios: 80:20, 60:40, 20:80 and, finally, pure ethanol. Cells in pure ethanol were dried using a critical-point drying device (Leica, EMCPD030) to preserve the morphology of the cells and the structure of the nanopillars. The SEM images of cells were obtained using a field-emission scanning electron microscope (FEI Nova 200). To investigate the cell-nanostructure interactions, the membranes of the cells on the nanopillars were removed by placing samples in a cytoskeleton buffer (CB; 150 mM NaCl, 5 mM EGTA, 5 mM MgCl2, 5 mM glucose, 10 mM MES, pH 6.1) at room temperature for a short time. The cells were then fixed and dried by critical-point drying as described above.

### AFM measurement

To calculate the rigidity of the nanopillar substrates, the apparent Young’s moduli of the nanopillars were measured with an atomic force microscope (AFM; JPK, Nanowizard II) using cantilevers (ULTRASHARP, MIKROMASCH) with a force constant of 0.1-0.4 N/m and a resonance frequency of 17–24 kHz. The rigidity was calculated using K = (3/64 πED^4^/L^3^), where K is the rigidity, E is the apparent Young’s modulus of the nanopillar, D and L are the diameter and the height of the nanopillars [41].
